# Global Research on Hemodialysis Nutrition and Patient-Centered Priorities: A Bibliometric Analysis (2006–2025)

**DOI:** 10.3390/healthcare14010028

**Published:** 2025-12-22

**Authors:** Chin-Huan Huang, Ming-Chi Lu, Malcolm Koo

**Affiliations:** 1 Department of Nutrition Therapy, Dalin Tzu Chi Hospital, Buddhist Tzu Chi Medical Foundation, Dalin, Chiayi 622401, Taiwan; 2 School of Medicine, Tzu Chi University, Hualien 970374, Taiwan; 3 Division of Allergy, Immunology and Rheumatology, Dalin Tzu Chi Hospital, Buddhist Tzu Chi Medical Foundation, Dalin, Chiayi 622401, Taiwan; 4 Department of Medical Research, Dalin Tzu Chi Hospital, Buddhist Tzu Chi Medical Foundation, Dalin, Chiayi 622401, Taiwan; 5 Dalla Lana School of Public Health, University of Toronto, Toronto, ON M5T 3M7, Canada

**Keywords:** hemodialysis, chronic kidney disease, nutrition, diet quality, oral nutritional supplements, plant-forward diet, gut microbiota, bibliometric analysis

## Abstract

**Background:** Optimal nutritional care is essential to improving outcomes in hemodialysis, yet translation of evidence into routine practice remains uneven across settings. To inform health system planning and implementation priorities, we mapped global research on hemodialysis-related nutrition. **Methods:** We searched the Web of Science Core Collection for English-language original articles on nutrition and hemodialysis from 1 January 2006 to 13 October 2025. Publication trends, productivity by country and institution, influential journals and authors, citation impact, and conceptual structure via Keyword Plus co-occurrence, trend, and thematic evolution analyses were assessed using the bibliometrix package (version 5.0) in R. **Results:** A total of 332 articles from 115 journals were identified, with substantial growth and multidisciplinary authorship, though international collaboration remains limited. The United States contributed 21.4% of publications and achieved the highest citation impact, while China, Japan, Iran, and Brazil formed the next tier of contributors. The *Journal of Renal Nutrition* accounted for 16.6% of papers. Highly cited studies established links between dietary intake, mineral and electrolyte management, and survival, while supporting the use of intradialytic oral nutritional supplements. Thematic evolution showed a shift from biochemical markers toward patient-centered priorities, including diet quality, adherence, body composition, mental health, and quality of life. Emerging directions point to whole-diet approaches and microbiome-modulating strategies. **Conclusions:** Global research on diet and hemodialysis has progressed from foundational nutrient studies to multidimensional, patient-focused approaches. Our findings suggest opportunities for health systems to strengthen dietitian-led models of care, integrate patient-reported outcomes, and prioritize scalable nutrition interventions within routine dialysis services.

## 1. Introduction

Chronic kidney disease (CKD) remains a major global health challenge, affecting over 800 million people and placing substantial pressure on healthcare systems due to its long-term clinical and service demands [1,2]. As kidney function declines to end-stage kidney disease (ESKD), most patients require kidney replacement therapy, with hemodialysis continuing to be the predominant modality worldwide, particularly in regions with limited transplant access [3]. The high prevalence of hemodialysis use and its intensive, ongoing care needs make it a critical focus for health-system planning and resource allocation.

Despite its life-extending benefits, hemodialysis introduces complex nutritional challenges that directly influence clinical outcomes and quality of life. Metabolic disturbances related to uremic toxins, chronic inflammation, and dialysis-associated nutrient losses create sustained barriers to maintaining adequate nutritional status [4]. Dietary restrictions aimed at managing phosphorus, potassium, sodium, and fluid balance often reduce dietary flexibility and energy intake, while elevated protein needs are frequently unmet in routine care [5]. These factors contribute to high rates of protein–energy wasting (PEW) (28–54% globally), which is strongly linked to hospitalization, mortality, and poorer patient-reported outcomes [6,7]. Optimizing nutritional care is therefore a core component of improving both survival and the lived experience of people receiving hemodialysis [8].

Evidence-based strategies exist to improve nutritional status in hemodialysis, including oral nutritional supplements [9], intradialytic nutrition support [10], dietary counseling interventions [11], and treatment adherence improvement programs [12]. Network meta-analyses suggest that dietary interventions are among the most effective non-pharmacological approaches for improving nutritional markers [13]. However, nutrition care is unevenly delivered across dialysis centers, with variability in access to dietetic services, integration of behavioral support, and routine use of patient-reported outcomes. Suboptimal nutrition contributes to higher healthcare utilization and increased costs of care for dialysis programs. These gaps suggest that generating evidence alone is insufficient; implementation within real-world dialysis services remains a central challenge.

Addressing these challenges requires the active engagement of the entire multidisciplinary ecosystem involved in hemodialysis care. This includes nephrologists focused on metabolic control and survival outcomes, renal dietitians who translate guidelines into practical medical nutrition therapy, and health administrators responsible for resource allocation. Most importantly, it involves the patients themselves, whose priorities often center on quality of life, symptom burden, and dietary autonomy rather than biochemical markers alone. Understanding how research trends align with the distinct needs of these stakeholders is essential for bridging the gap between scientific publication and clinical practice.

Although many trials and reviews have advanced knowledge in renal nutrition, less attention has been given to understanding how the research landscape has evolved, where evidence is concentrated, and which areas remain under-examined [14]. A comprehensive overview of publication trends, research priorities, and thematic evolution is needed to inform coordinated health-system planning, guide resource allocation, and identify opportunities to better align research with patient-centered care.

Bibliometric analysis offers a systematic approach to examining research patterns, collaboration networks, and thematic evolution across a field [15,16]. By mapping how evidence has developed over time, bibliometrics can support health-system decision-making by revealing research strengths, areas of duplication, and under-explored topics that warrant investment. This approach enables researchers, clinicians, and policymakers to align future studies with implementation needs and patient-centered priorities, rather than generating evidence in isolation.

A recent bibliometric analysis by Shakhshir et al. mapped global research trends on nutritional status and dialysis using the Scopus database, covering publications from 1952 to 2022 [17]. While the study provided a valuable overview of the field’s evolution, it had several notable limitations. It relied exclusively on Scopus and employed a title-only search strategy, which may have omitted relevant publications that used alternative terminology or were indexed in other databases. Furthermore, the analysis encompassed both hemodialysis and peritoneal dialysis populations, without distinguishing the unique nutritional challenges, metabolic alterations, and research developments specific to hemodialysis patients.

To address these gaps, we conducted a bibliometric analysis of hemodialysis-related nutrition research indexed in the Web of Science Core Collection from 2006 to 2025. This 20-year timeframe was selected to focus on contemporary trends relevant to current health system planning, distinct from historical biochemical foundational work. We sought to (1) map global publication patterns and identify leading countries, institutions, journals, and contributors; (2) examine citation impact and the characteristics of influential studies; and (3) analyze the conceptual structure and evolution of research themes. These objectives are intended to provide an updated overview of how evidence in hemodialysis nutrition has developed and to support clinicians selecting evidence-based interventions, researchers identifying areas requiring further inquiry, and policymakers planning nutrition support within dialysis services.

## 2. Materials and Methods

### 2.1. Data Source and Search Strategy

**Figure 1:**
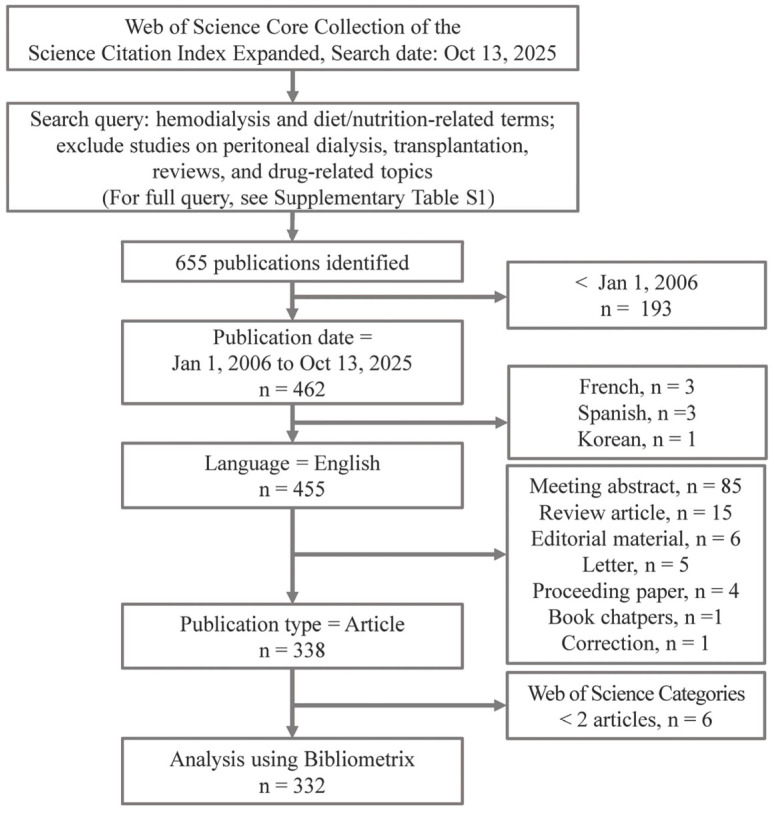
Study Flow Diagram.

A comprehensive literature search was conducted in the Science Citation Index Expanded of the WoS Core Collection to identify publications on hemodialysis and nutritional research (Figure 1). The search strategy, including the specific combination of proximity operators and exclusion criteria, was developed by the senior author and critically reviewed by the co-authors prior to data extraction. Due to the bibliometric design relying on metadata extraction, formal independent dual-screening of records was not performed. However, the final dataset was examined by the research team to ensure alignment with the inclusion criteria.

The WoS Core Collection was selected as the data source for this study to ensure consistency of bibliographic records. WoS provides structured fields for authors, affiliations, Keywords Plus, and cited references that are compatible with bibliometric software.

The search query was designed to retrieve original articles in English that examined the relationship between hemodialysis and nutritional research (Supplementary Table S1). The search included publications from 1 January 2006, to the date of the search, 13 October 2025. The search targeted titles containing the terms “hemodialysis” or “haemodialysis” in combination with dietary-related terms such as “diet*”, “nutrition*”, “oral nutrition”, “oral nutrition supplement*”, “intradialytic parenteral nutrition”, “dietary adherence”, “dietary protein”, “protein-energy wasting”, and dietary restrictions (e.g., sodium, potassium, phosphorus). These were further refined using proximity operators to capture relevant concepts within five words of intake, restriction, supplementation, counseling, education, adherence, or intradialytic context. To maintain focus on primary research related to hemodialysis, studies were excluded if they referenced peritoneal dialysis, kidney transplantation, or various forms of reviews and protocols. In addition, records mentioning pharmacological interventions, drug therapy, or specific medications in the abstract were excluded. These exclusion criteria were applied to minimize the inclusion of pharmaceutical efficacy trials (e.g., phosphate binder comparisons) which can dominate the literature volume, thereby ensuring the analysis remained focused on dietetic, lifestyle, and behavioral nutrition interventions. To further improve the relevance of the search, WoS Categories containing fewer than two articles were excluded from the final analysis.

### 2.2. Bibliometric Analysis

The dataset retrieved from the WoS was imported into RStudio (version 2024.12.0 Build 467) and analyzed using the bibliometrix package (version 5.0) in R [18]. Standard descriptive statistics, including absolute frequencies, percentages, and mean citation counts, were used to quantify trends in annual publication volume, geographic distribution, and institutional productivity. The annual publication growth rate was estimated using the Compound Annual Growth Rate (CAGR), which provides a smoothed measure of average yearly growth across the study period. CAGR was calculated using the standard expression: (Ending Value/Starting Value)^1/Number of Years^ − 1. In this analysis, the ending and starting values correspond to the number of publications in 2025 and 2006, respectively, and the number of years is the length of the observation period.

Country-level analysis was conducted using custom R code to ensure consistent geographic classification. We retained country labels as indexed in WoS (for example, preserving “Taiwan”), while standardizing others (such as converting “Russian Federation” to “Russia” and consolidating constituent countries of the United Kingdom). Total citations were taken directly from the WoS Core Collection export files (tag: TC) and aggregated without modification. KeyWords Plus are automatically generated indexing terms produced by Clarivate’s algorithm, which identifies frequently occurring terms in the titles of referenced articles to capture concepts relevant to the publication that may not appear in the author keywords.

To explore the conceptual structure of the field, co-occurrence analysis of WoS Keyword Plus was conducted. A synonym list and a stop-word list were applied to standardize terminology and remove noninformative terms (Supplementary Table S2 and File S1, respectively). In the co-occurrence network, nodes represent keywords, node size indicates frequency, link thickness reflects co-occurrence strength, and colors correspond to thematic clusters. Only WoS Keyword Plus terms occurring at least five times were included in the analysis. Networks were visualized using the Fruchterman–Reingold layout algorithm, and clusters were identified with the Walktrap community detection method, using the default four steps to balance local structural sensitivity and modularity stability. Co-occurrence matrices were normalized using fractional counting to account for variation in keyword frequency. Trend topic analysis used a sliding window approach to map the temporal emergence of frequently occurring terms. Thematic evolution was evaluated across three predefined periods (2006–2014, 2015–2021, 2022–2025) using the bibliometrix thematic evolution function, which applies normalized clustering to track the progression of themes across intervals.

## 3. Results

### 3.1. Overview and Publication Trends

Over the 2006–2025 period, 332 original articles from 115 sources addressing hemodialysis and nutrition were identified. Annual output rose at an average rate of 5.2%. The corpus contained 8999 cited references, or approximately 27 per article. Authorship patterns reflected extensive collaboration: among 1946 contributing authors, the mean was 7.23 co-authors per article; only 5 articles were single-authored (about 1.5%); and 54 articles (16.3%) involved international co-authorship. Taken together, these indicators suggest a maturing and progressively expanding field with increasing multi-author and cross-border contributions

**Figure 2:**
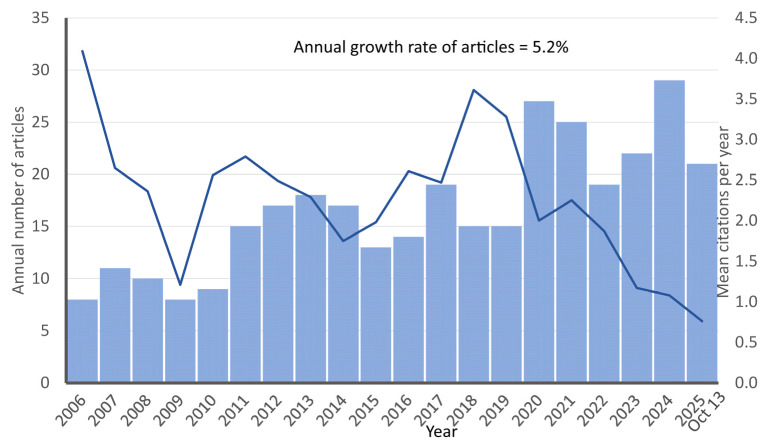
Trends in annual publication output and mean citation impact per year of original articles on nutritional research and hemodialysis published between 2006 and 2025 (N = 332). The bars represent the annual number of articles published (**left** axis), and the line represents the number of citations per year (**right** axis).

Annual output increased from 8 articles in 2006 to a peak of 29 in 2024, followed by 21 in 2025. Production was modest in 2006–2009 (8–11 papers per year), rose steadily during 2010–2014 (9–18), fluctuated in 2015–2019 (13–19, with 15 in both 2018 and 2019), and expanded from 2020 onward (27 in 2020, 25 in 2021, and 19–29 during 2022–2025). Mean citations per year were highest for the earliest cohort (4.09 in 2006), generally ranged around 2.0–2.8 during 2010–2017, reached local maxima in 2018–2019 (3.61 and 3.28), and then declined in the most recent years from 2.00 in 2020 to 0.76 in 2025. This downward trend is an expected bibliometric artifact reflecting citation accrual lag, as articles published more recently have had significantly less time to be read and cited than older works. It therefore does not indicate a decline in research impact (Figure 2).

### 3.2. Country Productivity

Table 1 summarizes the research productivity and citation impact of the top 10 countries contributing to original articles on nutritional research and hemodialysis from 2006 to 2025, based on the corresponding author’s affiliation. The results reveal that the United States is the leading contributor to nutritional research in hemodialysis, accounting for 71 articles, which constitutes 21.4% of the total publications in this field. The United States demonstrates a strong propensity for international collaboration, with 25.3% of its publications being multiple-country publications (MCPs). This high level of collaboration is mirrored in its research impact; publications with a corresponding author from the United States have amassed 2832 total citations, the highest among all countries, and maintain the highest mean citation rate of 3.9 citations per year.

Following the United States, China ranks second in volume with 29 articles (8.7%), but exhibits the lowest international collaboration rate among the top five at 3.5%. Japan follows closely with 28 articles (8.4%) and a moderate collaboration rate of 10.7%. Iran and Brazil, with 26 and 20 articles, respectively, show notable international collaboration rates of 15.4% and 10.0%. In terms of citation impact, Brazil and Iran have similar total citation counts (358 and 357, respectively), which are higher than those for China (260) and Japan (220). However, the mean citation rates for these four countries are clustered more closely together, ranging from 2.2 to 3.2, indicating a gap when compared to the citation impact of research led by the United States. Moreover, there appears to be a positive relationship between international collaboration and research impact; the United States leads in both metrics. However, this trend is not linear. China achieved the second-highest mean citation per year despite having the lowest international collaboration rate (3.5%) among the top five, suggesting that domestic research density and internal citation networks also play a significant role in impact accumulation.

### 3.3. Institutional Productivity

Table 2 presents the top five institutions contributing to original articles on nutritional research and hemodialysis published between 2006 and 2025.

### 3.4. Journal Productivity

Table 3 summarizes the five most productive journals publishing original articles on nutrition and hemodialysis between 2006 and 2025. A total of 115 journals contributed to the field, reflecting wide dissemination of research across diverse outlets. The mean output was approximately 2.9 articles per journal, suggesting that publication activity is concentrated within a small group of high-volume journals, while the majority published only one or two articles during the study period.

*The Journal of Renal Nutrition* was the most prolific source, with 55 articles, followed by *International Urology and Nephrology* (17), *Hemodialysis International* (14), *Nutrients* (13), and *Frontiers in Nutrition* (10). Journal productivity followed a right-skewed distribution consistent with Bradford’s law. According to this principle, when journals are ranked by their output on a specific topic, a small core of sources typically produces about one-third of all articles, while the remaining two-thirds are distributed across successively larger groups of journals in an approximate 1:n:n^2^ pattern. Our data align with this model: a compact core of highly productive journals accounted for roughly one-third of the publications, a second tier contributed a comparable share, and a broad periphery of journals published one or two papers each. This pattern indicates that, although several specialized journals serve as the primary platforms for dissemination, relevant research is also published in a wide range of nephrology, nutrition, and general medical journals.

### 3.5. Author Productivity

Table 4 presents the top five most prolific authors contributing to original articles on nutritional and hemodialysis between 2006 and 2025. The analysis identifies Kalantar-Zadeh, K. from the University of California as the most productive author with 17 articles. He is followed by Kovesdy, C. P. with nine publications and Kopple, J. D. with eight, both also affiliated with institutions in the United States. Due to ties in publication counts, the subsequent ranks are shared among multiple authors: Azar, A., Beberashvili, I., and Karupaiah, T. are tied for fourth place with seven publications each, while Daud, Z. A. M. and Sevick, M. A. share the fifth rank with six publications each.

In terms of research impact, Kalantar-Zadeh, K. also leads with the highest number of total citations (918), followed by Kopple, J. D. (696) and Kovesdy, C. P. (642). However, the mean citations per year indicate a sustained high impact for both Kovesdy, C.P. (5.8) and Kopple, J. D. (5.7). To provide a more refined measure of contribution that accounts for co-authorship, a fractionalized article score was calculated. “Articles fractionalized” assigns publication credit by dividing a single paper’s value equally among all its co-authors, thereby adjusting for collaboration levels. The fractionalized scores for all top authors were substantially lower than their total article counts, a consistent pattern indicating a high degree of collaboration across the field. For instance, the leading author’s 17 articles yielded a fractionalized score of 2.4, while other top authors had scores ranging from 0.7 to 1.2. This demonstrates that the work of these leading researchers is typically characterized by contributions to publications produced by large, multi-author teams.

### 3.6. Most Cited Articles

Table 5 presents the top 10 most cited original articles published on nutritional research and hemodialysis between 2006 and 2025. The collection is published in high-impact journals, including the *American Journal of Clinical Nutrition*, the *Journal of the American Society of Nephrology*, and the *American Journal of Kidney Diseases*. A significant portion of these seminal papers consists of large-scale observational and longitudinal studies that establish fundamental links between dietary factors and patient outcomes. These articles collectively investigate the prognostic value of key nutritional markers, including dietary intake of protein and potassium, serum levels of lipids, and sodium balance, consistently linking them to the critical clinical endpoints of mortality and survival. This focus demonstrates that the bedrock of highly cited research in this area has been the identification and validation of nutritional risk factors in the hemodialysis population.

Another prominent theme within this highly cited literature is the evaluation of specific nutritional interventions, particularly those administered during the dialysis treatment itself. Three of the top ten articles investigate the effects of intradialytic nutritional support, including both parenteral [19] and oral supplements [20,21], yielding pivotal findings for clinical practice. Notably, the second-ranked article is a prospective randomized study demonstrating that intradialytic parenteral nutrition did not improve survival in malnourished patients, a landmark finding that has influenced clinical guidelines [19]. Other high-impact studies in this cluster examined the benefits of oral supplements, showing improvements in physiological markers like protein homeostasis, thereby providing the evidence base for this common therapeutic strategy.

**Table 5:** Ten most-cited original articles on nutritional research and hemodialysis between 2006 and 2025.

Rank	Title, Publication Year [Reference Number]	Journal (2024 Journal Impact Factor)	Total Citations	Total Citations per Year	Normalized Total Citations
1	Concentrated red grape juice exerts antioxidant, hypolipidemic, and antiinflammatory effects in both hemodialysis patients and healthy subjects, 2006 [22]	*American Journal of Clinical Nutrition* (6.9)	252	12.6	3.1
2	Intradialytic parenteral nutrition does not improve survival in malnourished hemodialysis patients: A 2-year multicenter, prospective, randomized study, 2007 [19]	*Journal of the American Society of Nephrology* (9.4)	197	10.4	3.9
3	Association between serum lipids and survival in hemodialysis patients and impact of race, 2007 [23]	*Journal of the American Society of Nephrology* (9.4)	191	10.0	3.8
4	Longitudinal associations between dietary protein intake and survival in hemodialysis patients, 2006 [24]	*American Journal of Kidney Diseases* (8.8)	172	8.6	2.1
5	Dietary potassium intake and mortality in long-term hemodialysis patients, 2010 [25]	*American Journal of Kidney Diseases* (8.8)	148	9.2	3.6
6	Predialysis serum sodium level, dialysate sodium, and mortality in maintenance hemodialysis patients: The Dialysis Outcomes and Practice Patterns Study (DOPPS), 2012 [26]	*American Journal of Kidney Diseases* (8.8)	144	10.3	4.1
7	Comparative effects of dietary supplementation with red grape juice and vitamin E on production of superoxide by circulating neutrophil NADPH oxidase in hemodialysis patients, 2008 [27]	*American Journal of Clinical Nutrition* (6.9)	132	7.3	3.1
8	Intradialytic oral nutrition improves protein homeostasis in chronic hemodialysis patients with deranged nutritional status, 2006 [20]	*Journal of the American Society of Nephrology* (9.4)	128	6.4	1.6
9	Outcomes associated with intradialytic oral nutritional supplements in patients undergoing maintenance hemodialysis: A quality improvement report, 2012 [21]	*American Journal of Kidney Diseases* (8.8)	110	7.9	3.2
10	Prescribed dietary phosphate restriction and survival among hemodialysis patients, 2011 [28]	*Clinical Journal of the American Society of Nephrology* (7.1)	107	7.1	2.6

### 3.7. Web of Science Keyword Plus Co-Occurrence Network Analysis

**Figure 3:**
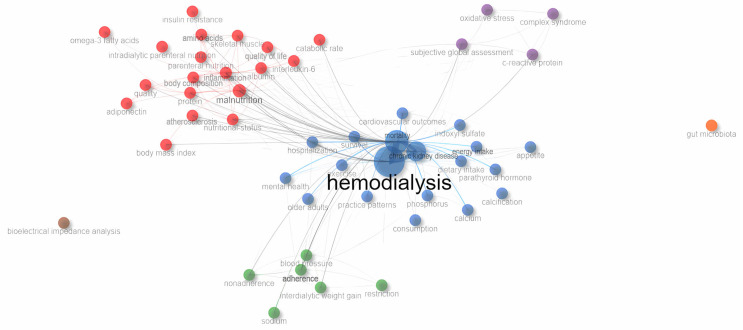
Co-occurrence network analysis of Web of Science Keyword Plus from original articles on nutritional research and hemodialysis between 2006 and 2025.

Figure 3 shows a visualization of co-occurrence network of WoS Keyword Plus of original articles on nutritional research and hemodialysis between 2006 and 2025. The co-occurrence network analysis of keywords illustrates a research landscape with several distinct and interconnected clusters. At the core of the network, “hemodialysis” and “chronic kidney disease” serve as the principal nodes, confirming their central role in this field of study. Emanating from this center is a large blue cluster that tightly integrates major clinical outcomes such as “mortality”, “hospitalization”, and “cardiovascular outcomes” with terms related to diet and mineral metabolism, including “dietary intake”, “phosphorus”, “calcium”, and “parathyroid hormone.” This demonstrates a primary research focus on the relationship between nutrient management, mineral bone disease, and survival endpoints in the hemodialysis population. A second significant cluster, colored red, is centered around “malnutrition”, and shows strong connections to “inflammation”, “albumin”, “body composition”, “skeletal muscle”, and “quality of life”, reflecting an investigative interest in assessing nutritional status and its functional and inflammatory consequences.

Moreover, a green cluster captures themes of treatment behavior and fluid balance, anchored by the terms “adherence”, “interdialytic weight gain”, and “blood pressure.” This represents a body of work focused on patient compliance and its direct physiological impacts. A purple cluster groups key inflammatory markers and assessment tools, including “C-reactive protein”, “oxidative stress”, and “subjective global assessment”, indicating a focus on diagnostic and prognostic screening. On the periphery of the network, terms with sparser connections suggest emerging or niche topics. Notably, “gut microbiota” appears as a nearly isolated node, signifying its novelty in the field, while “bioelectrical impedance analysis” forms a small, distinct cluster related to specific assessment techniques. Overall, the network map reveals a field organized around clinical outcomes and nutrient management, with strong sub-specialties in malnutrition, inflammation, and patient adherence.

### 3.8. Trend Topic Analysis of Web of Science Keyword Plus

**Figure 4:**
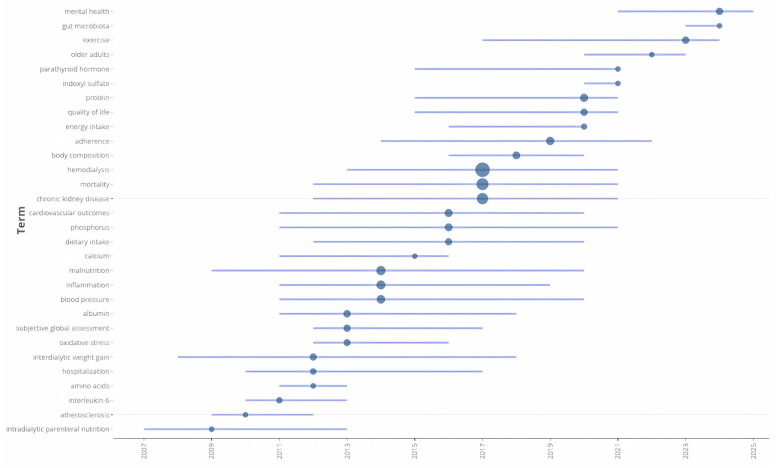
Trend topic analysis of nutritional research and hemodialysis between 2006 and 2025. Bubble size represents the relative frequency of each keyword, and horizontal bars indicate the active period during which the term appeared in publications.

Figure 4 presents a visualization of the trend of Web of Science Keyword Plus of original articles on nutritional research and hemodialysis between 2006 and 2025. Early research, primarily from 2007 to 2012, concentrated on fundamental clinical and biochemical aspects of hemodialysis. Keywords such as “intradialytic parenteral nutrition”, “atherosclerosis”, “interleukin-6”, and “amino acids” were prominent during this period, reflecting an emphasis on understanding the immediate nutritional and inflammatory consequences of renal failure and its treatment. This foundational phase also saw significant interest in established markers and complications such as “malnutrition”, “inflammation”, “blood pressure”, and mineral bone disease indicators such as “calcium” and “phosphorus”, which formed the core investigative landscape.

Beginning around 2015, a significant shift towards more patient-centered and holistic outcomes became apparent. Research interest expanded to include topics like “quality of life”, “body composition”, “adherence”, and the impact of “exercise.” More recently, since 2020, the focus has further diversified to encompass emerging areas and specific populations, as evidenced by the rise of keywords such as “older adults”, “mental health”, and “gut microbiota.” This progression indicates a move from investigating core pathophysiology and mortality towards a more comprehensive understanding of patient well-being, lifestyle interventions, and novel biological pathways in the context of chronic kidney disease.

### 3.9. Thematic Evolution Analysis of Web of Science Keyword Plus

**Figure 5:**
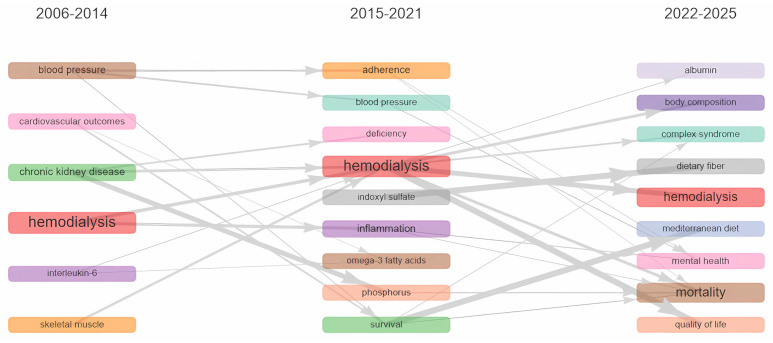
Thematic evolution analysis of nutritional research and hemodialysis between 2006 and 2025.

Figure 5 presents a visualization of thematic evolution of Web of Science Keyword Plus of original articles on nutritional research and hemodialysis between 2006 and 2025. In the initial period (2006–2014), the research landscape was defined by foundational themes such as “chronic kidney disease”, “cardiovascular outcomes”, and “blood pressure”, establishing the core clinical context of hemodialysis. As the field moved into the second period (2015–2021), these broad topics converged and evolved. “Hemodialysis” became a more dominant and central theme, while the research focus branched out into more specific areas. New themes emerged, including “inflammation”, mineral metabolism (“phosphorus”), patient behavior (“adherence”), and uremic toxins (“indoxyl sulfate”), indicating a shift from describing the general problems to investigating specific pathological mechanisms and treatment challenges. The broad theme of “cardiovascular outcomes” also began to consolidate into the more direct outcome measure of “survival.”

In the most recent period (2022–2025), the research landscape shows an evolution towards more patient-centered and holistic outcomes. The theme of “survival” from the previous period sharpened its focus to become “mortality.” A key new theme, “complex syndrome”, emerges directly from the central “hemodialysis” theme of the previous period, signifying a more integrated understanding of the overlapping pathologies in patients. Concurrently, there is an emergence of themes related to patient well-being, such as “quality of life” and “mental health”, which evolved from the earlier focus on “inflammation.” The research on diet has also matured, moving beyond single nutrients to encompass broader dietary patterns like “Mediterranean diet” and components such as “dietary fiber.” This final period illustrates a field that has progressed from identifying fundamental clinical issues to exploring specific mechanisms and is now focused on improving holistic, patient-reported outcomes and refining specific lifestyle interventions.

## 4. Discussion

This bibliometric analysis, based on the WoS Core Collection, shows that nutrition research in the hemodialysis setting has grown steadily over the past two decades, with expanding thematic scope and increasing contributions from diverse regions. The rise in publications suggests a broadening recognition of nutrition as an essential component of hemodialysis care and an area of continued clinical interest. While the United States leads in both output and citation impact, contributions from countries in Asia and South America indicate a gradual shift toward a more globally distributed evidence base. It is important to note, however, that these absolute publication counts do not account for economic disparities or research density per capita. Thus, the dominance of high-income nations likely reflects established research infrastructure and funding availability rather than solely disease burden. At the same time, international collaboration remains limited, which may influence the transferability and contextual relevance of findings across different health systems. Encouraging cross-country partnerships may support more adaptable research and help reflect the varied practice environments in which hemodialysis care is delivered.

Publications were concentrated in a core group of renal nutrition and nephrology journals, suggesting that research is reaching audiences with a clinical or nutrition focus. The *Journal of Renal Nutrition* contributed the largest share of publications, confirming its central role as the leading platform for renal nutrition research and its close alignment with the International Society of Renal Nutrition and Metabolism. *International Urology and Nephrology and Hemodialysis International* also served as key multidisciplinary venues bridging clinical nephrology with applied nutrition science. The increasing presence of hemodialysis nutrition studies in broader nutrition and chronic disease journals may support wider awareness and interdisciplinary exchange. From a service-delivery perspective, this expanding visibility may help inform approaches to nutrition care in dialysis settings, including dietitian-led counseling, intradialytic oral nutrition support, and routine nutritional screening. Although evidence describing these strategies continues to grow, their integration into everyday practice varies across regions and care models, reflecting differences in workforce, resources, and local priorities. Supporting context-appropriate adoption of nutrition care approaches may help improve the consistency of care provided to individuals receiving hemodialysis.

Authorship patterns in hemodialysis nutrition research reveal a small number of highly influential contributors and teams shaping key developments. For instance, Kalantar-Zadeh, K. from the University of California emerged as the most prolific and highly cited author, reflecting his leadership in malnutrition-inflammation and dietary management in kidney disease. While collaboration appears more common within countries than across regions, individual leadership can still elevate a country’s scientific visibility; notably, Karupaiah, T. and Daud, Z. A. M., two of the most productive authors, are affiliated with Malaysian universities, which were not otherwise top-ranked. The consistently low fractionalized article scores among these leading authors further indicate the cooperative nature of renal nutrition research, which increasingly relies on large, multidisciplinary teams to strengthen methodological rigor. While this concentration of expertise supports continuity and depth, broader participation is needed to diversify perspectives. Encouraging collaborative networks, especially those involving early-career researchers and institutions from under-represented regions, may support a more inclusive evidence base and facilitate knowledge exchange between health systems with varying resources. Future work could also consider study designs that explore implementation across diverse settings to better understand how nutrition care approaches translate into routine practice.

The most-cited studies in this field provide an empirical basis for integrating evidence-based nutrition care into hemodialysis service delivery. Large observational cohorts and randomized trials have clarified the survival implications of core dietary parameters, showing, for example, that an nPNA of 1.0 to 1.4 g/kg per day is associated with optimal outcomes, whereas intakes below 0.8 g/kg per day or above 1.4 g/kg per day increase mortality risk [24]. Analyses of the HEMO Study similarly challenge overly restrictive dietary practices. More stringent phosphate limits (≤870 mg per day) were linked to poorer nutritional status, while more liberal prescriptions were associated with lower all-cause mortality (hazard ratios 0.73 and 0.71) [28]. Additional work demonstrates that higher dietary potassium predicts greater five-year mortality [25], and that lower predialysis serum sodium (≤137 mEq/L) confers a significantly higher adjusted risk of death [26].

A parallel body of highly cited interventional research focuses on nutrition strategies that can be incorporated into routine dialysis care. Acute metabolic studies show that oral supplementation produces sustained anabolic effects, unlike intradialytic parenteral nutrition (IDPN) [20], and a randomized trial in malnourished patients found that early increases in prealbumin strongly predicted reduced two-year mortality, whereas adding IDPN to oral supplements did not improve outcomes [19]. The survival benefit associated with monitored intradialytic oral supplementation in a cohort of 28,611 hypoalbuminemic patients further demonstrates the scalability of oral nutrition programs within dialysis units [21].

Landmark research on lipid metabolism and inflammation expands the therapeutic scope. A large cohort study identified a survival paradox in which higher low-density lipoprotein (LDL) cholesterol correlated with improved overall survival, although LDL levels above 100 mg/dL nearly doubled cardiovascular mortality among Black patients [23]. Interventional studies using concentrated red grape juice provide additional mechanistic insight, demonstrating improvements in lipoprotein profiles and reducing oxidative stress markers more effectively than vitamin E supplementation alone [22,27].

Viewed collectively, these highly cited studies move the field beyond risk identification toward practical, patient-centered nutritional interventions that health systems can deploy at scale. Their findings support structured nutrition pathways, routine nutritional monitoring, and broader adoption of oral nutritional supplementation and individualized dietary counseling. These approaches are particularly relevant given persistent workforce constraints and the need for models of care that can deliver consistent, high-quality nutrition support across diverse dialysis settings.

The keyword co-occurrence network revealed an interconnected research structure focused on the relationship between nutrition, metabolic regulation, and clinical outcomes in hemodialysis. Central to this landscape is a body of work examining how dietary management and mineral metabolism relate to survival and cardiovascular health, reflecting the field’s clinical orientation. A prominent research domain centers on the interrelationship between malnutrition, inflammation, and body composition, emphasizing the functional and prognostic significance of nutritional status. Parallel to these biological dimensions, another conceptual stream addresses patient behavior and treatment adherence, particularly as they influence fluid management and overall well-being. In addition, a methodological cluster focusing on nutritional assessment tools and biomarkers illustrates the ongoing effort to refine diagnostic precision and monitor nutritional risk in clinical practice. Collectively, the co-occurrence patterns demonstrate that research in this field has evolved toward a multidimensional understanding of nutrition in hemodialysis, integrating clinical, behavioral, and functional perspectives.

The temporal evolution of research themes demonstrates a progression from biochemical and pathophysiological foundations to patient-centered perspectives. Early work (2006–2014) focused on core clinical parameters, such as cardiovascular outcomes, blood pressure, and mineral bone metabolism, reflecting efforts to characterize mortality risks and treatment complications. Between 2015 and 2021, these broad domains evolved into more specific and mechanistic themes, including inflammation, phosphorus metabolism, adherence, and uremic toxins, indicating a shift toward understanding modifiable pathways linking nutrition to outcomes. In the most recent period (2022–2025), the field has moved toward integrative themes such as “complex syndrome”, “quality of life”, and “mental health.” This conceptual shift signals a growing recognition of the multidimensional burden of hemodialysis and a more holistic view of nutritional health. As a result, increasing attention is being given to diet quality, psychosocial factors, and patient-reported outcomes. This shift is evidenced by the displacement of “mortality” and “calcium” as dominant nodes by “quality of life” and “mental health” in the most recent time quadrant (2022–2025). In the context of hemodialysis, this represents a transition to patient-centered priorities, where the goal of nutrition support extends beyond correcting biochemical derangements to preserving physical function and minimizing the burden of dietary restrictions. Future research should prioritize integrating patient-centered elements. This bibliometric trend aligns with the recent Kidney Disease: Improving Global Outcomes (KDIGO) 2024 [29] clinical guidelines, which advocate for shifting the goal of care from purely biochemical correction to the preservation of life participation and functional status.

Overall, the combined keyword and thematic analyses indicate a paradigm shift from nutrient-centric approaches to dietary and behavioral strategies. The emergence of topics such as “dietary patterns”, [30] “Mediterranean diet”, [31] and “dietary fiber” [32] points to increasing interest in whole-diet frameworks that promote overall diet quality while maintaining electrolyte balance. The growing yet still peripheral presence of gut microbiota research in the network reflects an important emerging frontier [33]. From a bibliometric standpoint, its topological separation indicates that this topic functions as a distinct sub-field rather than an integrated component of the mainstream hemodialysis literature. Unlike established clusters that are densely connected to outcomes such as mortality, the microbiome-related node has not yet converged with the core clinical evidence base. This separation is notable given the increasing attention to dysbiosis and the accumulation of gut-derived uremic toxins [34], such as indoxyl sulfate and p-cresyl sulfate, which have been implicated in inflammation, oxidative stress, and cardiovascular risk in hemodialysis patients. Despite this growing mechanistic insight, the current evidence base remains limited. Available meta-analyses are constrained by small trial sizes and substantial heterogeneity related to probiotic strains, dosages, and intervention durations, resulting in low certainty of evidence. The absence of standardized outcomes and the short duration of most studies further restrict the ability to determine the long-term clinical relevance of microbiome-targeted interventions. Together, these factors help explain the topic’s peripheral network position and indicate the need for more rigorous translational work. Future investigations should adopt harmonized protocols, incorporate mechanistic biomarkers of microbiome function, and evaluate patient-centered outcomes to establish more definitive evidence for integrating these interventions into hemodialysis care.

### 4.1. Strengths and Limitations

This study has several strengths. It provides an updated overview of hemodialysis nutrition research over nearly two decades, offering a broad perspective on publication patterns, thematic developments, and collaboration trends. The analysis reveals areas of concentration and emerging directions that may help inform future research planning. Furthermore, the study adheres to established reporting guidelines for bibliometric research [35,36] and ensures methodological transparency by explicitly detailing the search strategy, including the specific synonyms and stop words employed.

Nevertheless, this study has the inherent constraints of bibliometric research that should be considered. Only English-language journal articles indexed in the WoS Core Collection were included, which may affect the completeness and global representation of the evidence. However, WoS provides structured, high-quality metadata that ensures reproducibility and is widely accepted as a standard source for bibliometric studies in nutrition research. Moreover, we excluded pharmacotherapy-focused articles, which may have removed hybrid interventions that combine nutrition with medications.

In addition, our search strategy encompassed both enteral and parenteral nutrition strategies. While these are physiologically and clinically distinct interventions, both were included to capture the complete historical evolution of nutritional support research in hemodialysis, illustrating the temporal shift from intradialytic parenteral approaches to oral and dietary interventions.

### 4.2. Implications and Future Work

Regarding implications for practice, the identified citation landscape and conceptual clusters demonstrate the importance of a pragmatic, patient-centered approach that balances electrolyte management with adequate energy and high-quality protein intake. Routine use of intradialytic oral nutritional supplements, particularly for patients with hypoalbuminemia or insufficient intake, should be integrated into care, alongside dietitian-led education and behavior change strategies [11]. Nutrition education programs and digital support tools aimed at improving dietary adherence can be embedded into standard dialysis care and assessed using validated nutritional and patient-reported outcome measures [37]. A 2025 systematic review and meta-analysis indicated that digital health technologies-based interventions, specifically mobile applications and tele-nutrition platforms, resulted in significant improvements in serum albumin, hemoglobin, and electrolyte control (potassium and phosphorus) among hemodialysis patients [38]. These tools offer scalable solutions by facilitating real-time dietary monitoring (e.g., artificial intelligence-based diet recall), providing personalized feedback, and overcoming geographical barriers to care. By embedding these digital ecosystems into routine practice, clinicians can extend the reach of medical nutrition therapy beyond the dialysis center, thereby improving both adherence and clinical outcomes.

Regarding implications for research, our findings point to several priorities. First, randomized and pragmatic trials are needed to evaluate whole-diet prescriptions tailored for hemodialysis patients, such as Mediterranean-style [39] or plant-based diets [40], incorporating individualized potassium and phosphorus management, in comparison to standard care. Second, studies examining the gut–kidney axis should adopt longer follow-up periods, standardized toxin panels, and broader outcome measures, including quality of life, sleep quality, and symptom burden [41]. Third, interventions should combine medical nutrition therapy with strategies to enhance adherence, and explore scalable delivery models, such as digital platforms or intradialytic counseling [42]. Finally, international collaborations are essential to address region-specific dietary challenges, enhance external validity, and bridge the research collaboration gaps identified in this bibliometric analysis.

## 5. Conclusions

This review provides an updated overview of hemodialysis nutrition research over the past two decades, showing publication patterns, thematic developments, and collaboration trends. The findings suggest a gradual shift toward more holistic and patient-centered perspectives on nutritional care, reflecting growing recognition that nutrition influences both clinical outcomes and the lived experience of treatment. These observations may support efforts to align future research and service development with approaches that are relevant to routine dialysis care, considerate of diverse practice settings, and responsive to patient needs.

Looking ahead, continued attention to research gaps and collaborative opportunities may help strengthen the evidence base and support the integration of nutrition care into routine dialysis services. To translate these findings into practice, health systems should consider the implementation of scalable, dietitian-led care models. This includes leveraging hybrid protocols that combine routine intradialytic support with digital monitoring to ensure equitable access to nutrition therapy regardless of geographic barriers. Focusing on these priorities may help reinforce the value of nutrition within hemodialysis care and ultimately support improvements in patient well-being.

## Figures and Tables

**Table 1:** Top five countries contributing to original articles on nutritional research and hemodialysis from 2006 to 2025.

Rank	Country	Total Articles (%)	MCP %	Total Citation	Mean Citation/Year
1	United States	71 (21.4)	25.3	2832	3.9
2	China	29 (8.7)	3.5	260	3.2
3	Japan	28 (8.4)	10.7	220	2.2
4	Iran	26 (7.8)	15.4	357	2.7
5	Brazil	20 (6.0)	10.0	358	2.3

MCP: Multiple-Country Publication. MCP represents the number of articles where the corresponding author’s institution is collaborating with authors from at least one other country.

**Table 2:** Top five institutions contributing to original articles on nutritional research and hemodialysis published between 2006 and 2025.

Rank	Institution	Country	Total Articles
1	University of California System	United States	55
2	University of California Los Angeles	United States	38
3	University of California Los Angeles Medical Center	United States	28
4	Taipei Medical University	Taiwan	23
5	Shamir Medical Center (Assaf Harofeh)	Israel	22
5	Tel Aviv University	Israel	22

Six institutions are listed due to tied ranks in publication counts. System-level, campus-level, and subunit-level affiliations were retained as separate categories without hierarchical consolidation to strictly reflect the affiliations reported in the bibliographic records in the Web of Science metadata.

**Table 3:** Top five journals contributing to original articles on nutritional research and hemodialysis published between 2006 and 2025.

Rank	Journal	Number of Articles (%)	2024 Journal Impact Factor	Web of Science Category (Journal Impact Factor Quartile)
1	*Journal of Renal Nutrition*	55 (16.6)	3.2	Nutrition & Dietetics (2) Urology & Nephrology (1)
2	*International Urology and Nephrology*	17 (5.1)	1.9	Urology & Nephrology (3)
3	*Hemodialysis International*	14 (4.2)	1.2	Urology & Nephrology (3)
4	*Nutrients*	13 (3.9)	5.0	Nutrition & Dietetics (1)
5	*Frontiers in Nutrition*	10 (3.0)	5.1	Nutrition & Dietetics (1)

**Table 4:** Top five most productive authors contributing to original articles on nutritional research and hemodialysis between 2006 and 2025.

Rank	Author	Institution and Country	Total Articles	Articles Fractionalized	Total Citation	Mean Citation/Year
1	Kalantar-Zadeh, Kamyar	University of California (United States)	17	2.4	918	4.5
2	Kovesdy, Csaba P.	University of Tennessee Health Science Center (United States)	9	1.2	642	5.8
3	Kopple, Joel D.	University of California Los Angeles Medical Center (United States)	8	1.2	696	5.7
4	Azar, Ada	Shamir Medical Center (Israel)	7	0.9	94	3.3
4	Beberashvili, Ilia	Shamir Medical Center (Israel)	7	0.9	94	3.3
4	Karupaiah, Tilakavati	Taylor’s University (Malaysia)	7	0.8	192	3.0
5	Daud, Zulfitri Azuan Mat	Universiti Putra Malaysia (Malaysia)	6	0.7	192	3.0
5	Sevick, Mary Ann	New York University (United States)	6	0.7	189	3.6

Eight authors are listed due to tied ranks in publication counts.

## Data Availability

No new data were generated with this research.

## Supplementary Materials

The following supporting information can be downloaded at: https://www.mdpi.com/article/10.3390/healthcare14010028/s1. Table S1: Detailed search strategy for identifying records in the Web of Science Core Collection related to hemodialysis and dietary research (DOCX format). Table S2: Synonym list used in the bibliometric analysis (CSV format). File S1: Stop-word list used in the bibliometric analysis (TXT format).

## Abbreviations

The following abbreviations are used in this manuscript:

CKD	Chronic kidney disease
ESKD	End-stage kidney disease
IDPN	Intradialytic parenteral nutrition
LDL	Low-density lipoprotein
MCP	Multiple-country publication
NADPH	Nicotinamide adenine dinucleotide phosphate
nPNA	Normalized protein nitrogen appearance
PEW	Protein–energy wasting
WoS	Web of Science
