# Supplementary material for: Global Research on Hemodialysis Nutrition and Patient-Centered Priorities: A Bibliometric Analysis (2006–2025)
Source: Healthcare (Basel). 2025 Dec 22;14(1):28. doi: 10.3390/healthcare14010028 (PMC12785684; doi:10.3390/healthcare14010028)
Supplement: Supplementary file 1 [file healthcare-14-00028-s001.zip › Supplementary Table S1.pdf]

Supplementary Table S1. Detailed search strategy for identifying records in the Web of Science Core Collection related to hemodialysis and dietary research

(
(TI=(hemodialysis OR haemodialysis)
AND
(TI=((diet* OR nutrition* OR "oral nutrition" OR "oral nutrition supplement*" OR "intradialytic parenteral nutrition" OR "dietary adherence" OR "dietary protein" OR "protein-energy wasting" OR "sodium restriction*" OR "potassium restriction*" OR "phosphorus restriction*") NEAR/5 (intake OR restriction OR supplement* OR counsel* OR educat* OR adherence OR intradialytic OR oral))
OR
AB=((diet* OR nutrition* OR "oral nutrition" OR "oral nutrition supplement*" OR "intradialytic parenteral nutrition" OR "dietary adherence" OR "dietary protein" OR "protein-energy wasting" OR "sodium restriction*" OR "potassium restriction*" OR "phosphorus restriction*") NEAR/5 (intake OR restriction OR supplement* OR counsel* OR educat* OR adherence OR intradialytic OR oral))
))
NOT
(
TS=("peritoneal dialysis" OR peritoneal OR transplant* OR meta-analys* OR meta analy* OR "systematic review" OR "scoping review" OR "umbrella review" OR "narrative review" OR "overview of reviews" OR bibliometric* OR guideline* OR consensus OR protocol*)
OR
AB=(drug* OR pharmac* OR medication* OR pharmaco* OR "drug therapy" OR "pharmacologic*" OR antihypertens* OR "ace inhibitor*" OR "angiotensin receptor blocker*" OR ARB* OR "beta-blocker*" OR "beta blocker*" OR "calcium channel blocker*" OR diuretic* OR statin* OR "hmg-coa reductase inhibitor*" OR "phosphate binder*" OR calcimimetic* OR "vitamin d analog*" OR erythropoies* OR ESA* OR "intravenous iron" OR "iron sucrose" OR anticoagulant* OR antiplatelet*)
OR
AB=(aldosterone OR atenolol OR lisinopril OR losartan OR valsartan OR enalapril OR ramipril OR amlodipine OR nifedipine OR diltiazem OR verapamil OR metoprolol OR carvedilol OR clonidine OR hydralazine OR sevelamer OR lanthanum OR "calcium acetate" OR "calcium carbonate" OR "ferric citrate" OR "sucroferric oxyhydroxide" OR cinacalcet OR etelcalcetide OR calcitriol OR paricalcitol OR alfacalcidol OR epoetin OR darbepoetin OR "methoxy polyethylene glycol-epoetin" OR atorvastatin OR rosuvastatin OR simvastatin)
))
